# Differentiating mega cisterna magna from cisterna magna arachnoid cysts: The diagnostic utility of quantitative diffusion‐weighted imaging parameters

**DOI:** 10.1002/acm2.70166

**Published:** 2025-07-14

**Authors:** Quanxiang Li, Zhe Feng, Haichao Cheng, Xin Cao, Yaping Ge, Hao Shi, Jun‐Ying Wang

**Affiliations:** ^1^ Department of Radiology TCM Hospital of Yiyuan County Yiyuan County Shandong China; ^2^ Department of Radiology Huashan Hospital Fudan University Shanghai China; ^3^ Department of Radiology The First Affiliated Hospital of Shandong First Medical University & Shandong Province Qianfoshan Hospital Jinan Shandong China

**Keywords:** cisterna magna arachnoid cyst, differential diagnosis, diffusion‐weighted imaging, mega cisterna magna, temporal arachnoid cyst

## Abstract

**Objective:**

To explore the utility of diffusion‐weighted imaging (DWI)‐derived apparent diffusion coefficient (ADC) and exponential apparent diffusion coefficient (eADC) parameters in differentiating mega cisterna magna (MCM) from cisterna magna arachnoid cysts (CMAC).

**Methods:**

Retrospective analysis of MRI data from 40 MCM patients, 46 CMAC patients (confirmed via clinical follow‐up and imaging criteria), and 36 temporal arachnoid cysts (TAC) as controls was performed. Independent sample *t*‐tests were used to compare ADC and eADC values among the three groups.

**Results:**

The eADC values of MCM, CMAC, and TAC were (3.75 ± 0.27) × 10^−2^, (4.53 ± 0.54)×10^−2^, and (4.35 ± 0.30) ×10^−2^ mm^2^/s, respectively. There was no significant difference in the eADC value between CMAC and TAC (*p* = 0.191). However, the eADC value of MCM was significantly different from the two groups (*p* = 0.000). The ADC values of MCM, CMAC, and TAC were (3.30 ± 0.08) × 10^−3^, (3.11 ± 0.12) ×10^−3^, and (2.97 ± 0.71) × 10^−3 ^mm^2^/s, respectively. Significant differences in ADC were observed between MCM and both CMAC and TAC (*p* = 0.000/0.003).

**Conclusion:**

DWI‐derived ADC and eADC parameters can effectively differentiate mega cisterna magna from cisterna magna arachnoid cysts.

## 1 INTRODUCTION

Mega cisterna magna (MCM) is a congenital condition characterized by an enlarged cisterna magna, in which the cerebellar cortex or vermis is located more than 10 mm from the inner occipital plate.^1^ Intracranial arachnoid cysts are cystic lesions that develop between the brain parenchyma and the arachnoid membrane. Arachnoid cysts within the cisterna magna are termed cisterna magna arachnoid cysts (CMAC).^2^

Although most MCM and CMAC cases are asymptomatic, some individuals may experience symptoms such as headache, dizziness, or localized brain dysfunction. Clinically, symptomatic patients often require surgical intervention, with a specific surgical approach depending on the nature of the lesion.^3^ However, differentiation between MCM and CMAC may pose challenges with conventional imaging modalities.

Several techniques have been employed to treat sylvian arachnoid cysts, including cysto‐peritoneal shunting and cyst marsupialization.^4^ For MCM, conservative or non‐surgical approaches are generally preferred.^5^ Precise differentiation between MCM and CMAC is critical for guiding clinical decision‐making, particularly in determining the necessity of surgical intervention.

Early studies proposed CT cisternography (CTC), MRI, and phase‐contrast (PC) cine MRI for differentiating these conditions. However, CTC is invasive and limited by contrast agent allergies and extravasation risks.^6^ PC cine MRI is also limited by factors such as heart rate, scanning angle, and long scan times.^7^

**FIGURE 1:**
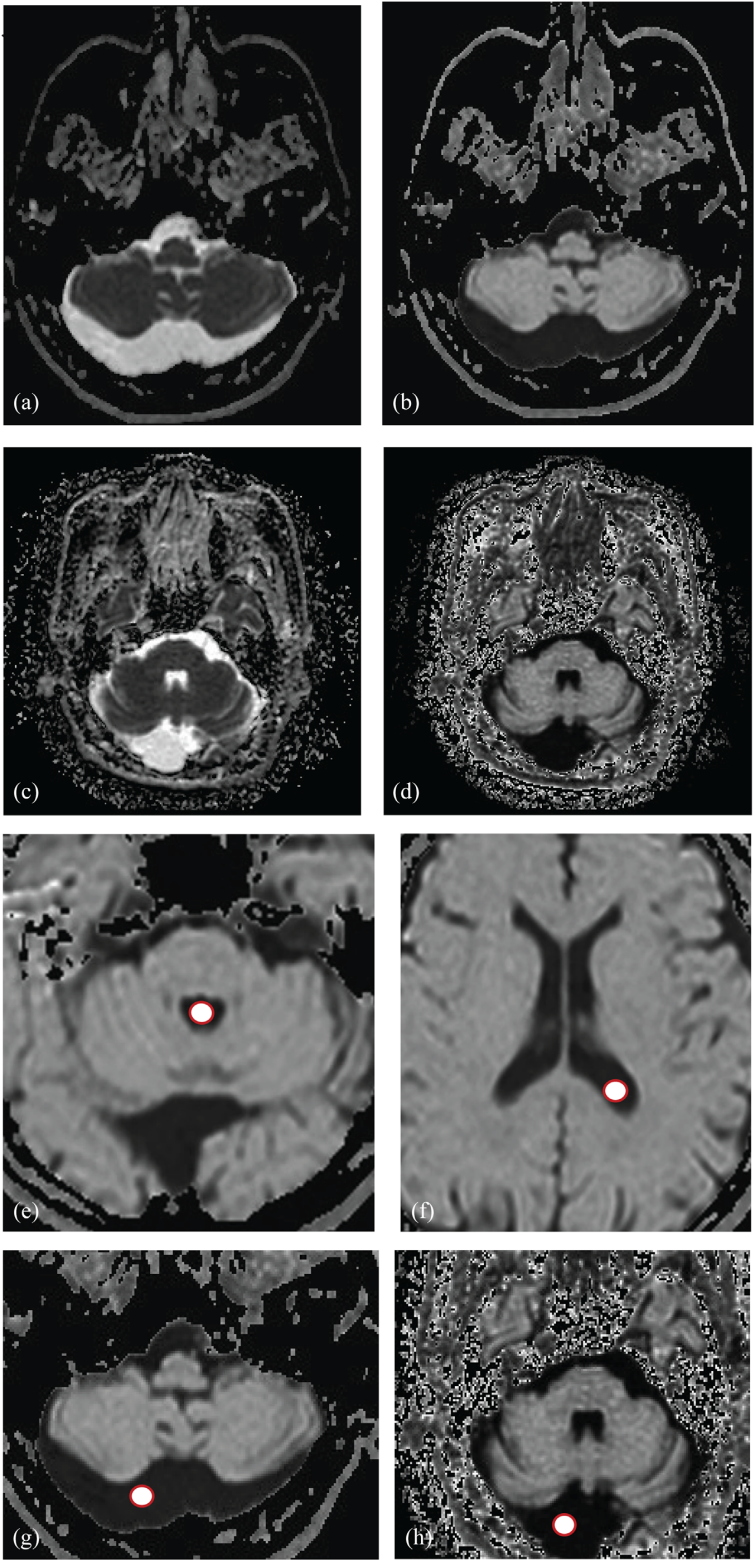
(a,b) Axial ADC (a) and eADC (b) maps of a 52‐year‐old male with MCM. (c,d) Axial ADC (c) and eADC (d) maps of a 65‐year‐old female with CMAC. (e–h) The red circle delineates the ROI placement in the ventricle and lesion. ADC, apparent diffusion coefficient; CMAC, cisterna magna arachnoid cyst; eADC, exponential apparent diffusion coefficient; MCM, mega cisterna magna; ROI, region of interest.

Diffusion‐weighted imaging (DWI) is a powerful imaging technique that assesses the diffusion of water molecules within tissues. By analyzing DWI data, two key parameters can be derived: the apparent diffusion coefficient (ADC) and the exponential apparent diffusion coefficient (eADC). ADC is calculated from DWI data and quantifies the Brownian motion of water molecules in biological tissues. The ADC value reflects the magnitude of diffusion restriction, with lower values indicating reduced water mobility. The formula is: ADC =$\;\frac{ln\;\frac{S_{1}}{S_{2}}}{b_{2}-b_{1}}\;$, where (*b*_1_) and (*b*_2_) represent applied diffusion gradient strengths, and *S*_1_ and *S*_2_ are the corresponding DWI signal intensities from the same region of interest (ROI). eADC is derived from the signal intensity ratio between high‐ and low‐*b*‐value DWI acquisitions: eADC = e^(‐b*ADC)^.^8^ This parameter is designed to mitigate the T2 shine‐through effect inherent in conventional DWI by normalizing signal differences independent of ADC. Unlike its name suggests, eADC does not represent an exponential transformation of ADC but rather a direct signal normalization strategy (Figure 1a–d). These parameters provide adequate quantitative information about the degree of water molecular diffusion within tissues.

DWI has established utility in pathological characterization and oncological grading.^9^ In general, the high signal intensity of a lesion indicates limited diffusion within the lesion. However, certain conditions, such as the T2 shine‐through effect, can confounded by this interpretation. The eADC map is a more advanced technique that can mitigate the influence of T2 transmission. It directly reflects signal changes caused by the diffusion behavior of the lesion itself, providing a more accurate measure of diffusion restriction.

Although eADC has been widely used in the diagnosis of abdominal diseases and the evaluation of prostate cancer,^10^ there is currently no research exploring potential differences in CSF dynamics and arachnoid cysts.

This study systematically investigated the ADC and eADC values between MCM and CMAC. By quantifying microstructural differences in water molecule diffusivity, these parameters offer a novel framework for characterizing CSF dynamics, potentially improving diagnostic precision in cases requiring differential diagnosis.

## 2 MATERIAL AND METHODS

### 2.1 Subjects

This study was approved by the institutional review board, with all participants providing written informed consent. Between 2019 and 2024, 121 patients were prospectively enrolled.  Cases were categorized into three groups by two senior neuroradiologists with 20 and 30 years of diagnostic experience, respectively:

1. **Group A (MCM)**: 40 patients (21 male/19 female; mean age: 55 ± 12 years); Primarily incidentally detected during routine physical examinations. Three patients presented with dizziness.
2. **Group B (CMAC)**: 45 patients (20 male/25 female; mean age: 45 ± 15 years); Clinical manifestations included dizziness (12), headache (8), sensorimotor deficits (4), hydrocephalus (4), epilepsy (2), hypertension (1), visual impairment (1), and brain abscess (1).
3. **Group C (TAC control)**: 36 patients (20 male/16 female; mean age: 40 ± 14 years); Majority diagnosed via clinical evaluation and imaging, with headache as the primary symptom in a subset.

All cases were evaluated according to predefined imaging criteria, with ambiguous presentations excluded from analysis (Flow Chart 1). “Ambiguous cases” refer to lesions demonstrating indistinct imaging features that prevent definitive differentiation between MCM and CMAC. In such instances, two board‐certified neuroradiologists reached interobserver discrepancy (IOD) regarding lesion classification. To ensure diagnostic homogeneity and prevent misclassification bias, these cases were excluded from analysis.

**FLOW CHART 1:**
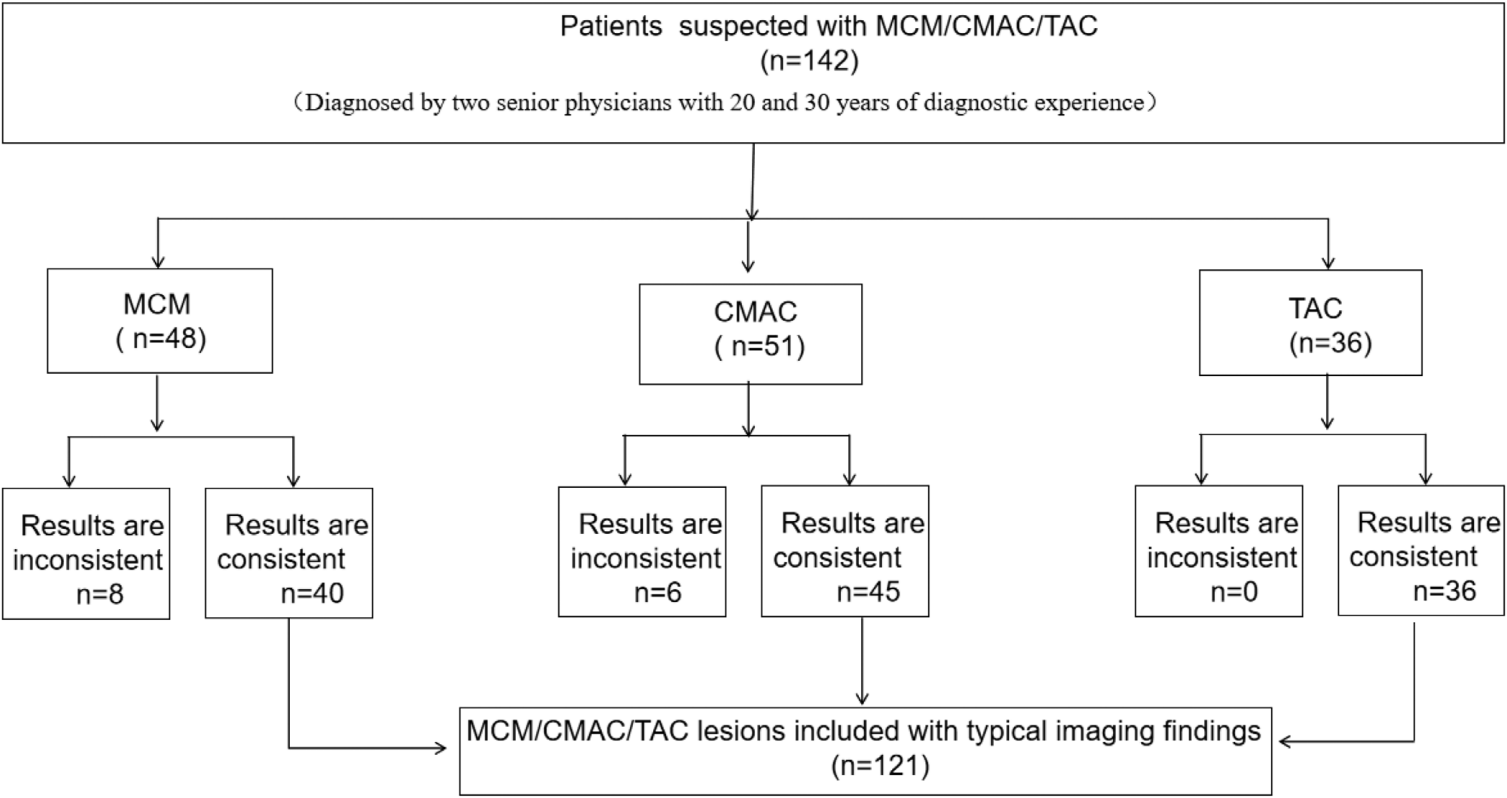
Patient selection. *n*: Number of cases; MCM: Mega cisterna magna; CMAC: cisterna magna arachnoid cyst; TAC: temporal polar arachnoid cyst.

### 2.2 Imaging acquisition

All MRI examinations were conducted on a 3.0T Discovery MR750 scanner (GE Healthcare, USA) equipped with an 8‐channel phased‐array head coil. Selected patients underwent additional computed tomography (CT) or contrast‐enhanced MRI. Image acquisition included axial, coronal, and sagittal orientations. ADC and eADC maps were generated using dedicated workstation software (Advantage Workstation 4.2, GE Healthcare) and analyzed offline. Patients were positioned supine and underwent T1‐weighted imaging (T1WI), T2‐weighted imaging (T2WI), fluid‐attenuated inversion recovery (FLAIR), and DWI sequences (*b* = 0, 1000 s/mm^2^). Some patients also underwent CT and contrast‐enhanced MRI. Scans were acquired in axial, coronal, and sagittal planes. ADC and eADC maps were generated and analyzed using dedicated workstation software (ADW4.2). The ADC value and eADC value of CSF in the lesion area, lateral ventricle, and fourth ventricle were measured.

### 2.3 Data analysis

Two neuroradiologists with ≥10 years of experience independently reviewed imaging and clinical data for MCM, CMAC, and TAC groups in adherence to standardized imaging protocols. To account for interindividual variability in cerebrospinal fluid (CSF) signal characteristics, quantitative measurements of ADC and eADC were obtained from: 1. Lesion core (MCM/CMAC) or cystic cavity (TAC); 2. Contralateral lateral ventricles; 3. Fourth ventricles. ROI placement followed strict guidelines: 3–5 mm^2^ regions positioned centrally in fluid spaces, avoiding structural boundaries (cyst walls, choroid plexus, ventricular margins) (Figure 1e–h). Intraindividual comparisons were performed between lesion and ventricular CSF for MCM and CMAC groups. Additionally, intergroup comparisons (MCM vs. CMAC, MCM vs. TAC, CMAC vs. TAC) were conducted using TAC as the control cohort. Statistical significance was evaluated using the Mann–Whitney U test for non‐parametric data, with *p* < 0.05 considered significant.

### 2.4 Statistical analysis

Statistical analysis was performed using SPSS 22.0 and GraphPad 8.0. Measurement data are presented as mean ± standard deviation. A paired‐sample *t‐*test was used to compare CSF ADC and eADC values between the fourth and third ventricles in the MCM, CMAC, and TAC groups. Independent samples *t*‐test was performed to compare ADC and eADC values ​​between the three groups. A *p* value < 0.05 was considered statistically significant.

## 3 RESULTS

### 3.1 Typical imaging features of MCM and CMAC

**FIGURE 2:**
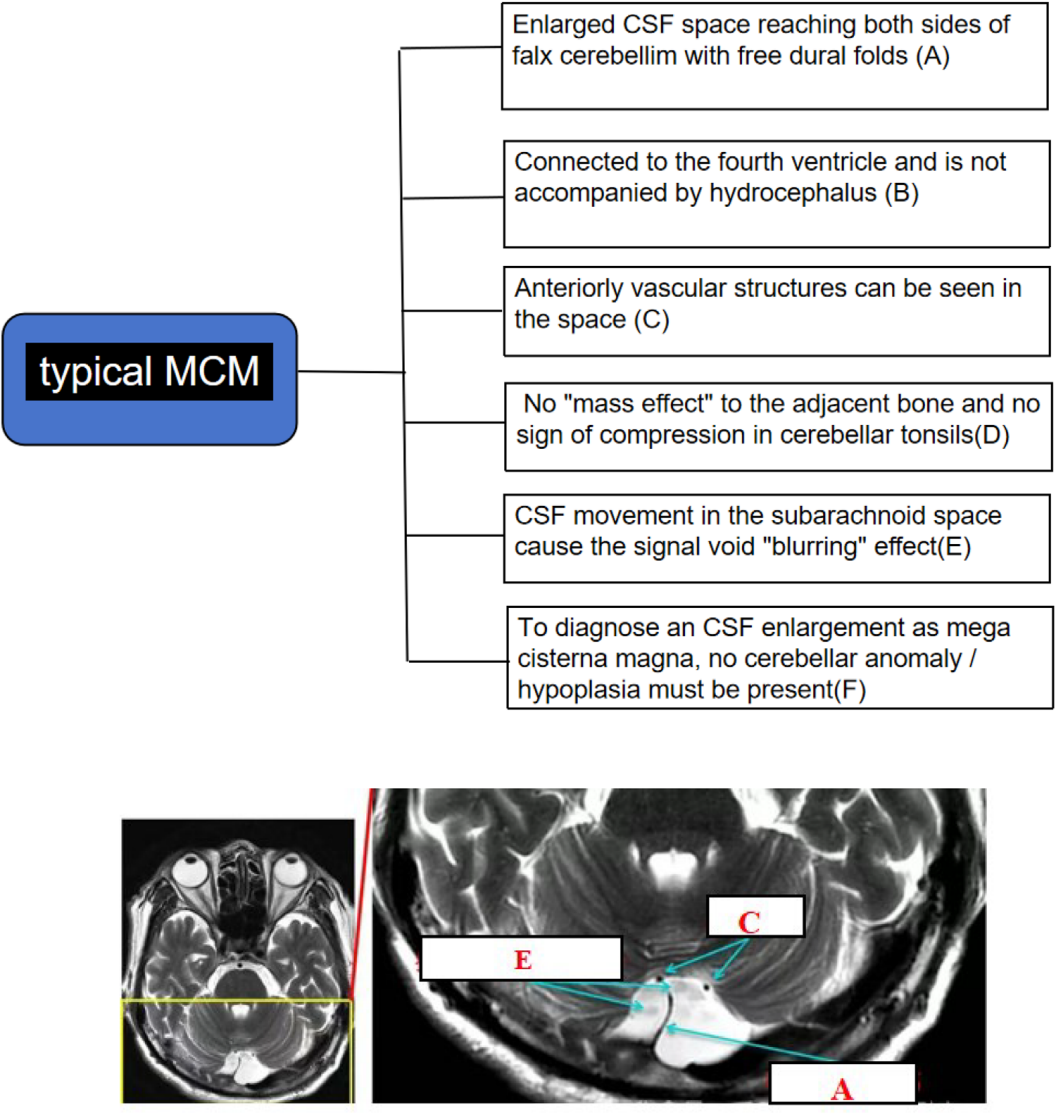
A patient with MCM, 52‐year, male, arrow head indicates typical imaging feature (A.C.E). MCM, mega cisterna magna.

**FIGURE 3:**
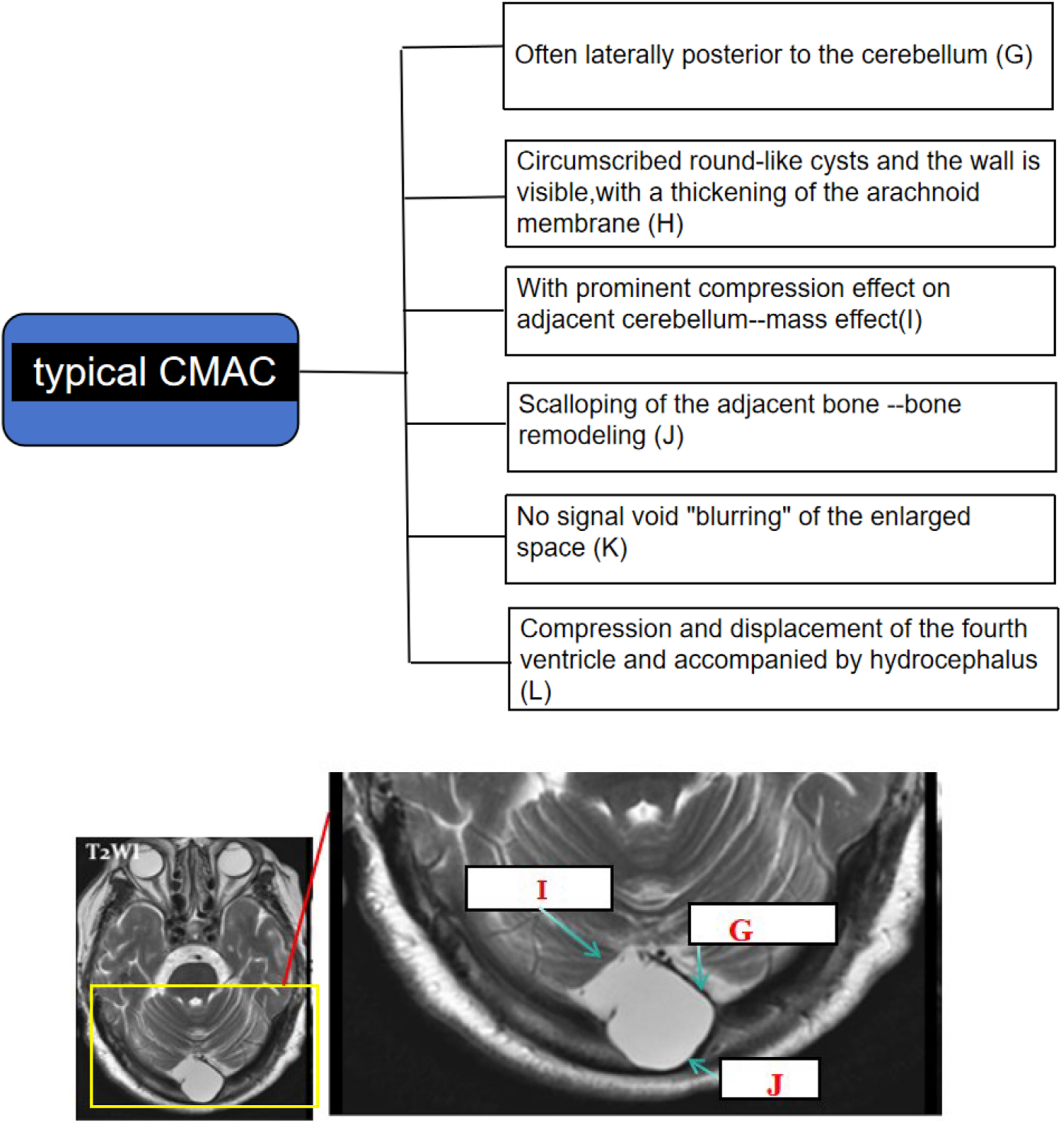
Patient with CMAC, 65‐year, female, complaining of dizziness, arrow head indicates typical imaging feature (I.G.J). CMAC: cisterna magna arachnoid cyst.

Building on prior investigations and current findings, this study synthesizes the differential diagnostic criteria between cisterna magna and arachnoid cysts as follows (Figures 2 and 3).

### 3.2 Cerebrospinal fluid (CSF) diffusion metrics

**FIGURE 4:**
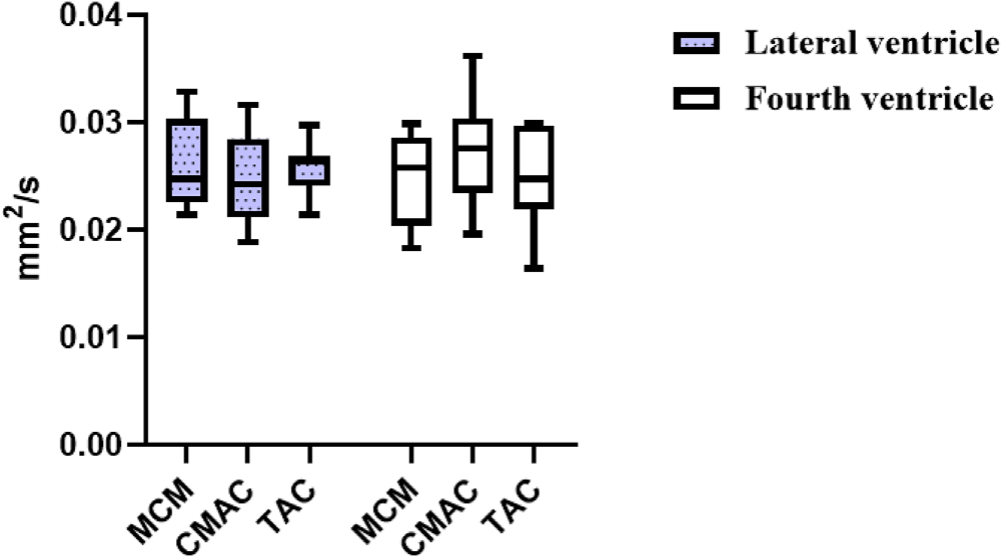
eADC metrics of lateral ventricular CSF in three cohorts (MCM, CMAC, TAC). ADC, apparent diffusion coefficient; eADC, exponential apparent diffusion coefficient; MCM, mega cisterna magna; CMAC, cisterna magna arachnoid cyst; TAC, temporal polar arachnoid cyst. CSF, cerebrospinal fluid. 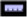
Indicates eADC values of lateral ventricular CSF. 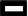
Indicates eADC values of fourth ventricular CSF.

**FIGURE 5:**
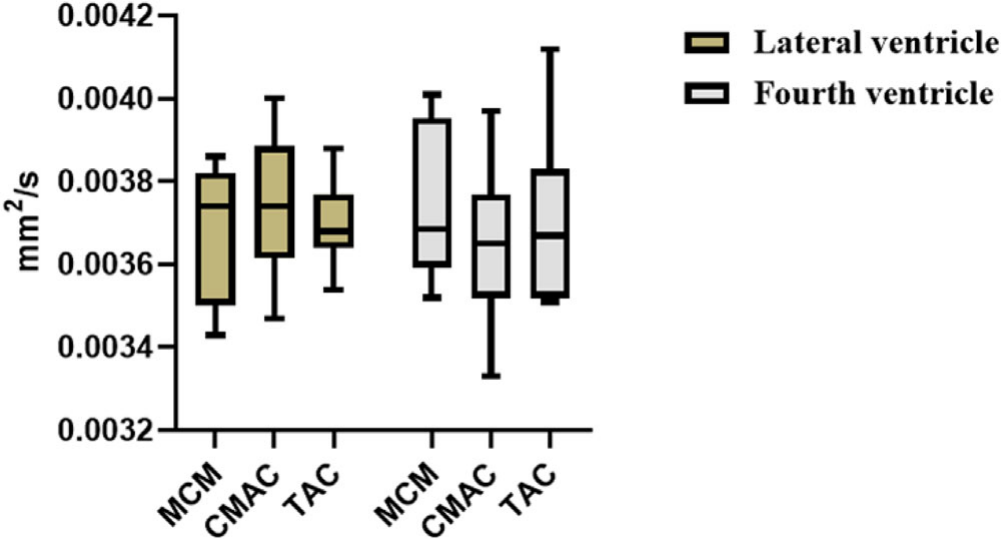
ADC Metrics of lateral ventricular CSF in three cohorts (MCM, CMAC, TAC). ADC, apparent diffusion coefficient; eADC, exponential apparent diffusion coefficient; MCM, Mega cisterna magna; CMAC, cisterna magna arachnoid cyst; TAC, temporal polar arachnoid cyst. CSF, cerebrospinal fluid. 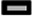
Indicates ADC values of lateral ventricular CSF. 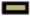
Indicates eADC values of fourth ventricular CSF.

To minimize interindividual variability, standardized CSF measurements were acquired from lateral and fourth ventricles in all groups (Table 1). ADC and eADC values were obtained from these compartments using consistent region‐of‐interest (ROI) placement protocols. Statistical analysis (Figures 4 and 5) revealed no significant differences in CSF ADC or eADC values among MCM, CMAC, and TAC groups in either ventricular compartment (*p *= 0.00).

**TABLE 1:** Differences of ADC and eADC values among MCM, CMAC, and the CSF.

	MCM	CSF	*p*	CMAC	CSF	*p*
**ADC** (mm^2^/s)*10^−3^	3.30 ± 0.08	3.70 ± 0.09	0.00	3.11 ± 0.12	3.74 ± 0.16	0.00
**eADC** (mm^2^/s) *10^−2^	3.75 ± 0.27	2.57 ± 0.21	0.00	4.53 ± 0.54	2.49 ± 0.41	0.00

Abbreviations: ADC, apparent diffusion coefficient; CMAC, cisterna magna arachnoid cyst; CSF, Cerebrospinal fluid; eADC, exponential apparent diffusion coefficient; MCM, Mega cisterna magna.

### 3.3 Intergroup comparisons with TAC control

Using temporal arachnoid cysts (TAC) as the control cohort, intergroup comparisons were performed between TAC and both MCM/CMAC groups (Table 2). No significant differences in ADC or eADC were observed between TAC and CMAC. Conversely, TAC demonstrated statistically significant differences in both eADC (*p *= 0.00) and ADC (*p *= 0.03) compared to MCM, as determined by Mann–Whitney U test.

**TABLE 2:** Difference of ADC and eADC values among MCM, CMAC, and TAC.

	MCM		CMAC	
	ADC (mm^2^/s) *10^−3^	eADC (mm^2^/s) *10^−2^	ADC (mm^2^/s) *10^−3^	eADC (mm^2^/s) *10^−2^
	3.30 ± 0.08	3.75 ± 0.27	3.11 ± 0.12	4.53 ± 0.54
**TAC**	2.97 ± 0.71	4.35 ± 0.30	2.97 ± 0.71	4.35 ± 0.30
***P***	0.030	0.000	0.436	0.191

Abbreviations: ADC, apparent diffusion coefficient; CMAC, cisterna magna arachnoid cyst; CSF, Cerebrospinal fluid; eADC, exponential apparent diffusion coefficient; MCM, mega cisterna magna; TAC, temporal polar arachnoid cyst.

### 3.4 MCM versus CMAC diffusion metrics

**FIGURE 6:**
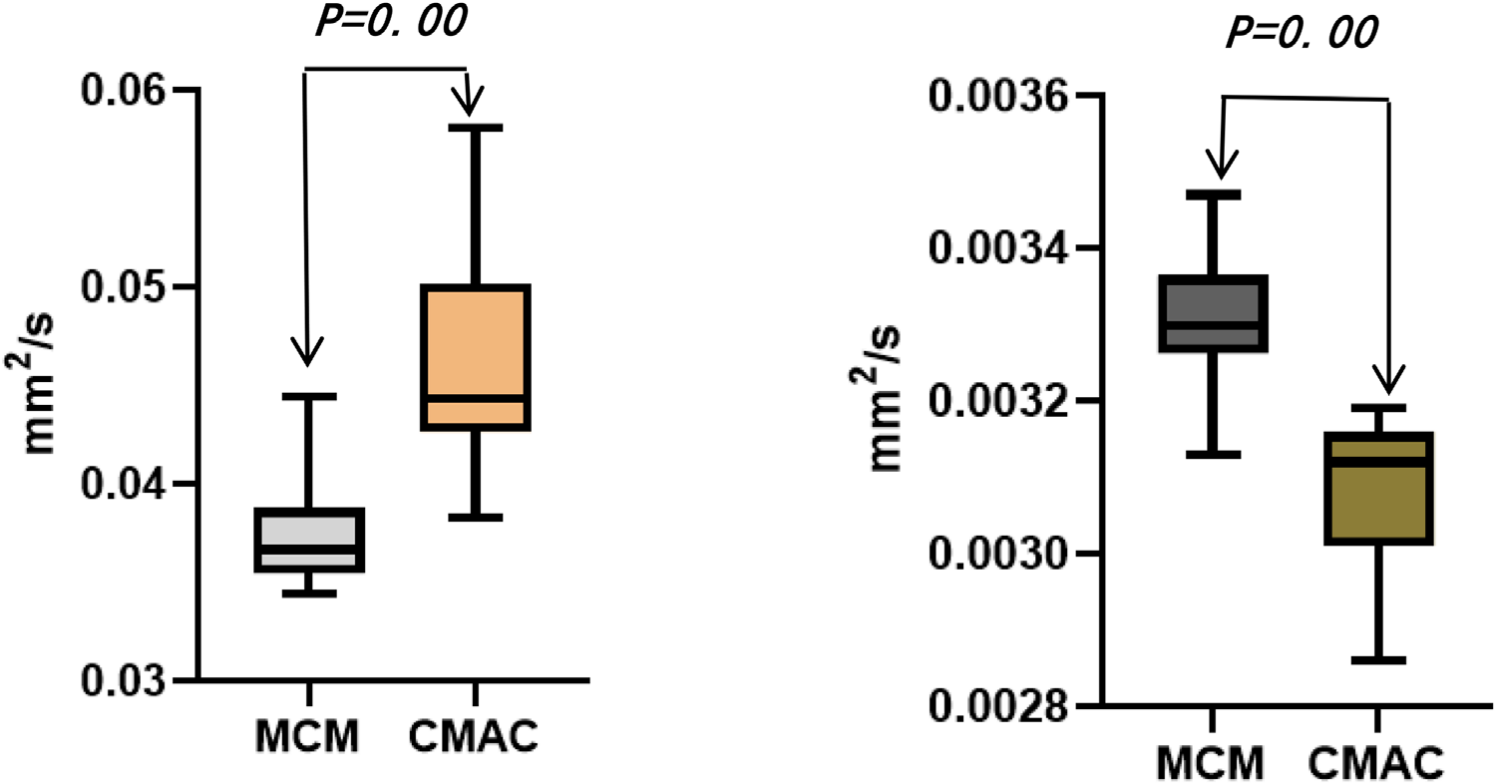
MCM versus CMAC diffusion metrics. CMAC, cisterna magna arachnoid cyst; MCM, mega cisterna magna.

MCM demonstrated significantly higher ADC values (*p* = 0.00) and lower eADC values (*p* = 0.00) compared to CMAC (Figure 6).

## 4 DISCUSSION

In this study, we systematically evaluated the DWI‐derived parameters, ADC and eADC, in MCM and CMAC. By ensuring high inter‐rater reliability and accounting for individual differences, we found statistically significant differences in ADC and eADC values between MCM and CMAC. Additionally, the ADC and eADC values of MCM were significantly different from those of CMAC and TAC. Furthermore, the eADC and ADC values of both MCM and CMAC were statistically different from those of CSF within the same group. Therefore, we concluded that DWI‐derived metrics, ADC and eADC, are robust tools for differentiating between MCM and CMAC.

Building on prior investigations,^11^, ^12^, ^13^, ^14^ and the current study's observations, we summarize the differential diagnosis between cistern magna and arachnoid cysts. Although it is readily distinguishable between a typical arachnoid cyst and cisterna magna, atypical cases may pose diagnostic challenges. Some arachnoid cysts may present with clinical symptoms like headache and dizziness due to increased intracranial pressure, and may even require surgical intervention in special cases. Accurate differentiation between MCM and CMAC is crucial for appropriate clinical management.

This study identified significant intergroup differences in ADC and eADC parameters between MCM and CMAC (*p* = 0.00). Pathophysiologically, MCM is hypothesized to represent either a chromosomal malformation or a common physiological variant characterized by cerebellomedullary cistern enlargement with unrestricted communication to the medullary subarachnoid space and CSF circulation.^15^ Conversely, CMACs are encapsulated CSF collections classified into three pathological subtypes based on communication with the subarachnoid space: closed, well‐communicating, and poorly communicating.^16^, ^17^ The observed diffusion differences likely reflect microstructural variations in fluid dynamics. MCM's open CSF communication allows partial water molecule exchange, whereas CMACs' encapsulated architecture restricts diffusion. These nanoscale differences, imperceptible on conventional imaging, are quantitatively captured by ADC and eADC maps, providing novel insights into retrocerebellar CSF compartmentalization.

DWI, a functional MRI technique sensitive to Brownian motion of water molecules, has established utility in cerebral ischemia assessment and brain tumor differentiation. In this study, standard DWI‐derived parameters apparent diffusion coefficient (ADC) and exponential ADC (eADC) were employed. MCM exhibited significantly higher ADC values compared to CMAC, while CMAC demonstrated higher eADC values, indicating greater T2 shine‐through effect mitigation. These findings suggest restricted water diffusivity in CMAC relative to MCM. However, eADC remains less validated for clinical use in differentiating posterior fossa CSF collections compared to ADC, warranting further investigation.

Additionally, as the control group, TAC showed no significant ADC/eADC differences compared to CMAC. Conversely, MCM demonstrated distinct ADC values compared to both arachnoid cyst groups, validating the microstructural differences in water diffusivity between communicating (MCM) and encapsulated (CMAC/TAC) CSF collections. These findings support the utility of TAC as a control cohort for arachnoid cyst studies, reinforcing the diffusion‐based differentiation between cisterna magna variants and true cysts.

Both MCM and CMAC demonstrated significantly lower ADC values compared to intraventricular CSF within the same group. This suggests restricted water diffusivity in both entities, contradicting prior assumptions that MCM represents freely circulating CSF. Although CMACs exhibit confined fluid spaces, MCMs show intermediate restriction, likely due to partial communication with the CSF circulation. This microstructural distinction may explain the observed compression effects on surrounding structures, as stagnant fluid fractions accumulate in non‐communicating regions. To our knowledge, this represents the first report quantifying diffusion restriction in retrocerebellar CSF collections.

A key limitation of this study is the absence of histopathological confirmation for MCM and CMAC, as these benign entities rarely necessitate surgical intervention, resulting in most of the MCM and CMAC without pathologically confirmed results. However, ambiguous cases were excluded from this study. All the included cases were selected from the more typical cases under the double‐blind evaluation of two senior doctors. In addition, after reviewing much literature, it was found that there are few reports on the two types of lesions, the literature referenced is generally older, and there is a lack of citations for new studies.

## 5 CONCLUSION

Typical arachnoid cysts and cisterna magna are readily distinguishable based on imaging features. For atypical cases requiring surgical intervention due to symptomatic presentation, ADC and eADC parameters demonstrate robust diagnostic performance in differentiating mega cisterna magna (MCM) from cisterna magna arachnoid cysts (CMAC).

## AUTHOR CONTRIBUTIONS

Quanxiang Li and Zhe Feng designed and performed the research, and wrote the paper. Jun‐Ying Wang designed the research and supervised the report; Yaping Ge and Xin Cao contributed to the analysis; Haichao Cheng and Hao Shi provided clinical advice.

## CONFLICT OF INTEREST STATEMENT

The authors declared no potential conflicts of interest with respect to the research, authorship, and/or publication of this article.

## ETHICS STATEMENT

All procedures performed in studies involving human participants were approved by the appropriate ethics committee of the First Affiliated Hospital of Shandong First Medical University.

## Data Availability

The data that support the findings of this study are available on request from the corresponding author. The data are not publicly available due to privacy or ethical restrictions.
